# Participation in Social Activities and Relationship between Walking Habits and Disability Incidence

**DOI:** 10.3390/jcm10091895

**Published:** 2021-04-27

**Authors:** Osamu Katayama, Sangyoon Lee, Seongryu Bae, Keitaro Makino, Ippei Chiba, Kenji Harada, Yohei Shinkai, Hiroyuki Shimada

**Affiliations:** 1 Department of Preventive Gerontology, Center for Gerontology and Social Science, National Center for Geriatrics and Gerontology, 7-430 Morioka-cho, Aichi, Obu City 474-8511, Japan; sylee@ncgg.go.jp (S.L.); bae-sr@ncgg.go.jp (S.B.); kmakino@ncgg.go.jp (K.M.); ichiba@ncgg.go.jp (I.C.); harada-k@ncgg.go.jp (K.H.); yshinkai@ncgg.go.jp (Y.S.); shimada@ncgg.go.jp (H.S.); 2 Japan Society for the Promotion of Science, 5-3-1 Koji-machi, Chiyoda-ku, Tokyo 102-0083, Japan

**Keywords:** social activity, walking habits, disability, older adults

## Abstract

Identifying the relationship between physical and social activity and disability among community-dwelling older adults may provide important information for implementing tailored interventions to prevent disability progression. The aim of this study was to determine the effect of the number of social activities on the relationship between walking habits and disability incidence in older adults. We included 2873 older adults (mean age, 73.1 years; SD, ±5.9 years) from the National Center for Geriatrics and Gerontology—Study of Geriatric Syndromes. Baseline measurements, including frequencies of physical and social activities, health conditions, physical function, cognitive function, metabolic parameters, and other potential disability risk factors (for example, the number of years of education); monthly assessment for disability was monitored through long-term care insurance certification for at least 2 years from baseline. During a mean follow-up of 35.1 months (SD, 6.4 months), 133 participants developed disability. The disability incidence was 19.0 and 27.9 per 1000 person-years for participants who walked more (≥3 times per week) and less (≤3 times per week) frequently, respectively. The potential confounding factor-adjusted disability hazard ratio was 0.67 (95% confidence interval, 0.46 to 0.96; *p* = 0.030). The relationship between habitual walking and the number of social activities was statistically significant (*p* = 0.004). The reduction of disability risk by walking was greater among participants with fewer social activities. Habitual walking was associated with disability incidence, with a more pronounced effect among older adults who were less likely to engage in social activities.

## 1. Introduction

Many developed countries have rapidly aging populations; Japan has the fastest aging population. As of 2020, 35.9 million people were aged over 65 years, which constituted 28.4% of the global population and the highest proportion globally [1]. Japan is expected to have the largest proportion of older adults worldwide by 2050 when 39.9% of the national population is projected to be aged over 65 years [1]. In developed nations facing an aging population, including Japan, many of these older adults require care [1,2]. Since the introduction of Japan’s long-term care insurance (LTCI) system in 2000, the number of older adults requiring LTCI service has increased. The Japanese LTCI system has operated for approximately 20 years and currently serves nearly 6.4 million people [3]. Dementia, cerebrovascular disease, and age-related weaknesses have been identified as the main causes of disability in older Japanese adults of both sexes.

Currently, the coronavirus disease (COVID-19) pandemic is spreading globally. A study conducted among older adults before (January 2020) and during (April 2020) the first wave of COVID-19 outbreaks in Japan showed that the duration of physical activity in older adults decreased by approximately 30% [4]. Similarly, the number of steps taken by older Japanese adults decreased by up to 30% after the initial spread of COVID-19 [5], and there are concerns that the incidence of disability may increase after the convergence of COVID-19 due to a decrease in daily physical activity [4]. Recently, the World Health Organization (WHO) has developed new guidelines on physical activity and sedentary behavior [6]. The guidelines provide evidence-based public health recommendations concerning the amount (frequency, intensity, duration) and types of physical activity that offer significant health benefits and mitigate health risks [6]. Walking is one of the most preferred and recommended activities that can be easily incorporated into daily life and maintained into old age [7]. Older adults should perform varied multicomponent and moderate- to vigorous-intensity physical activities that emphasize functional balance and strength training, at least three times a week, to enhance functional capacity and prevent falls [8].

Interestingly, a previous study indicated that the physical activity time was recovered after the spread of COVID-19 (June 2020) up to pre-infection (January 2020), although it was more difficult to recover when living alone and being socially inactive [9]. More attention is being paid to social activity in later life. Social activities are characterized by interactions with the environment and ingroup members, getting together with like-minded people, and the engagement of mind and body [10]. The government of Japan has indicated that in an aging society with diverse values, it will promote and support the participation of older adults in opportunities that enrich the spirit and fulfill a sense of purpose in life through social activities [11]. Previous studies have shown that participation in social activities is associated with a reduced risk of developing disability, dementia, and depression in the future, thereby maintaining the health of older adults [12,13,14,15]. Therefore, participation in social activities may reduce the risk of disability by modulating the relationship between physical activity and disability. Although it is well established that social and physical activities are each associated with the incidence of disability [14,16], it is unclear whether participation in social activity can influence the relationship between walking habits and the incidence of disability.

This means that identifying the relationship between physical and social activity and disability among community-dwelling older adults may provide important information for implementing tailored interventions to prevent disability progression. This study aimed to determine how participation in social activities is associated with the relationship between walking habits and disability incidence. We hypothesized a greater reduction in the risk of disability by walking habits among persons with fewer social activities compared with those with a higher number of social activities.

## 2. Materials and Methods

### 2.1. Design, Setting, and Participants

This was an observational prospective cohort study of adults enrolled in a population-based cohort study, which is part of the National Center for Geriatrics and Gerontology—Study of Geriatric Syndromes (NCGG–SGS). The NCGG-SGS is a cohort study with a primary goal to establish a screening system for geriatric syndromes and to validate evidence-based interventions for preventing geriatric syndromes [17]. This study investigated the association between walking habits and social activity participation at baseline and the incidence of disability during a mean follow-up of 35.1 months (standard deviation (SD), 6.4 months) from baseline.

Individuals who were aged ≥60 years or older, lived in Takahama City, were not hospitalized, were not in residential care, were not certified by the LTCI system as having a functional disability, or were not participating in another study (*n* = 9716) were sent an invitation letter to participate in the Takahama study. A total of 4167 community-dwelling older adults participated in the assessments, including face-to-face interviews and physical and cognitive function evaluation. We included participants who resided in Takahama city and were aged ≥60 years or older at the time of the study (September 2015–February 2017). We excluded participants who met the following criteria: (1) age < 65 years (*n* = 777); (2) with health problems (dementia, Parkinson disease, and stroke) (*n* = 240), such as dementia, Parkinson’s disease, and stroke, which have a direct impact on physical activity and disability incidence, based on information obtained by a qualified nurse who interviewed the participants face-to-face; (3) who needed support or care—as certified by the Japanese public LTCI system—due to disability (*n* = 75); (4) with disabilities affecting basic activities of daily living (ADLs) (*n* = 5); and (5) responses with missing variables of exclusion criteria (*n* = 197). Of the initial 4167 participants, 1294 were excluded based on these criteria. The study participants were followed up from September 2015 to February 2019, with a mean follow-up of 35.1 months (SD, 6.4 months). All participants provided written informed consent prior to participating in this study. The study protocol was approved by the Ethics Committee of the National Center for Geriatrics and Gerontology (No. 1440).

### 2.2. Disability Determination

Participants were tracked monthly for a new incidence of LTCI certification, as recorded by the Japanese LTCI system and measured by each municipal government. The LTCI system classifies a person in “Support Level 1 or 2” to indicate a need for assistance to support ADLs, or in “Care Levels 1 through 5” to indicate a need for continuous care [18]. In this study, disability was defined as any LTCI certification level, and we defined disability onset as the point at which a participant received LTCI certification.

### 2.3. Measurement of Physical and Social Activity

The physical and social activities were assessed from the activities listed in a self-reported questionnaire. Physical activity was assessed at baseline by asking participants about their participation in the following activities during the past year: walking, cycling, jogging, swimming, muscle training, yoga, gymnastics, dancing, hiking, playing golf, playing grand golf, or ball exercise. Exercise frequency was assessed by participants as never, once a month or less, several times a month, 1–2 times per week, 3–6 times per week, and every day [19]. In this study, persons who exercised at least 3 times a week were classified as exercising regularly, based on the WHO guidelines [6]. At baseline, walking had the highest percentage of participants who were “exercising regularly” among the 12 aforementioned physical activities (Supplementary Table S1). Therefore, only walking was included in the statistical analysis. Social activity was assessed at baseline by asking participants about their participation in the following group activities involving two or more people during the past year: officer of a senior club or neighborhood association, attending a regional event, engaging in environmental beautification activities, teaching, supporting activity, working, singing karaoke, dining out, partying with friends, shopping with friends, talking to friends (including phone calls), attending an event or concerts, or traveling. The frequency of participation in social activities was also assessed by participants as: never, once a month or less, several times a month, 1–2 times per week, 3–6 times per week, and every day [19]. In this study, individuals except those who did not participate at least once a year were classified as regular participants. Additionally, we calculated the average number of the 12 social activities wherein all participants took part. We then divided the participants into two groups: those with fewer social activities and those with more numbers of social activities, based on the average number of activities.

### 2.4. Potential Confounding Factors

Potential confounders included demographic variables, chronic disease, psychological factors, and metabolic parameters associated with disability in older adults. Therefore, our model included the following covariates: age at enrollment, sex, medication use, the presence of chronic diseases, cohabitation status, the number of years of education, grip strength, Mini-Mental State Examination (MMSE) [20] score, body mass index (BMI), total serum protein, glycated hemoglobin (HbA1c), and walk score. The presence of the following self-reported chronic diseases was also included among the covariates: heart disease, diabetes, hyperlipidemia, and spinal diseases. Grip strength was measured using a Smedley-type handheld dynamometer (GRIP-D; Takei Scientific Instruments Co., Ltd., Niigata, Japan) under strictly standardized conditions, using the same device, to avoid inter-observer and inter-device variability. We recorded one measurement of grip strength of each participant’s dominant hand, with participants in a standing position and having their elbows extended. We utilized Walk Score™ (Front Seat Management, Limited Liability Company (LLC), Seattle, WA, USA) [21], a publicly available website found to be valid and reliable for estimating accessibility to amenities within a comfortable walking distance [22]. The Walk Score uses data provided by the Google™ AJAX Search application program interface [23], along with a geography-based algorithm to identify nearby amenities and calculate a “walkability” score [21] based on the distance to amenities. Neighborhood walkability is associated with participation in physical and social activities by older adults [24,25,26]. The Walk Scores are associated with the number of steps per day, minutes of leisure walking, and daily moderate- to vigorous-intensity physical activities [27,28,29]. The Walk Score was calculated for all participants using their home addresses. Blood samples were taken at least 4 h after meals and were analyzed using standard laboratory techniques.

### 2.5. Statistical Analysis

One-way analysis of variance and Pearson’s chi-square tests were used to compare variables among groups of participants who were disability-free, with a disability, and who died or relocated. Similarly, the aforementioned tests were used to compare variables among participants who walked ≥3 times per week and participants who walked <3 times per week groups. Adjusted standardized residuals >1.96 indicated *p* < 0.05. Sensitivity analyses were performed to evaluate whether a potential bias could be introduced by the censoring mechanism for persons who died or relocated. We calculated cumulative incident disability during follow-ups for each of the two above-mentioned walking groups using the Kaplan–Meier curves. Intergroup differences were estimated using log-rank tests. Crude and adjusted Cox proportional hazard models were constructed to calculate hazard ratios (HR) with 95% confidence intervals (CI) for incident disability risk. Model 1 is a crude model. Model 2 is adjusted for age and sex. The factors associated with the incidence of disability among older Japanese have been shown to differ by age and sex [11,30]. In previous studies examining factors associated with disability incidence in older Japanese, models that adjust for age and sex have been used in many cases [14,16,31,32]. Model 3 is adjusted for the covariates in Model 2 and years of education, heart disease, diabetes, hyperlipidemia, spinal diseases, cohabitation status, BMI, total serum protein, HbA1c, medication, MMSE score, grip strength, and walk score. Given the significant effect of social activity participation on disability, we performed sub-analyses by applying Cox proportional hazard models to participants both in fewer and more social activities (number of participations <mean and ≥mean, respectively) groups separately. The significance level was set at *p* < 0.05. All analyses were performed using IBM SPSS, version 25.0 (IBM Japan, Tokyo, Japan).

## 3. Results

The final analysis included data from 2873 older adults (1618 women; mean age, 73.1 years; SD, 5.9 years; age range, 65–96 years). Of 2873 participants, 2693, 133, and 47 participants remained disability-free, developed a disability, and either died or relocated, respectively. Table 1 shows the baseline characteristics of the study participants who remained disability-free, developed a disability, and died or relocated from the study. Table 2 shows the baseline characteristics of study participants by walking levels.

**Figure 1:**
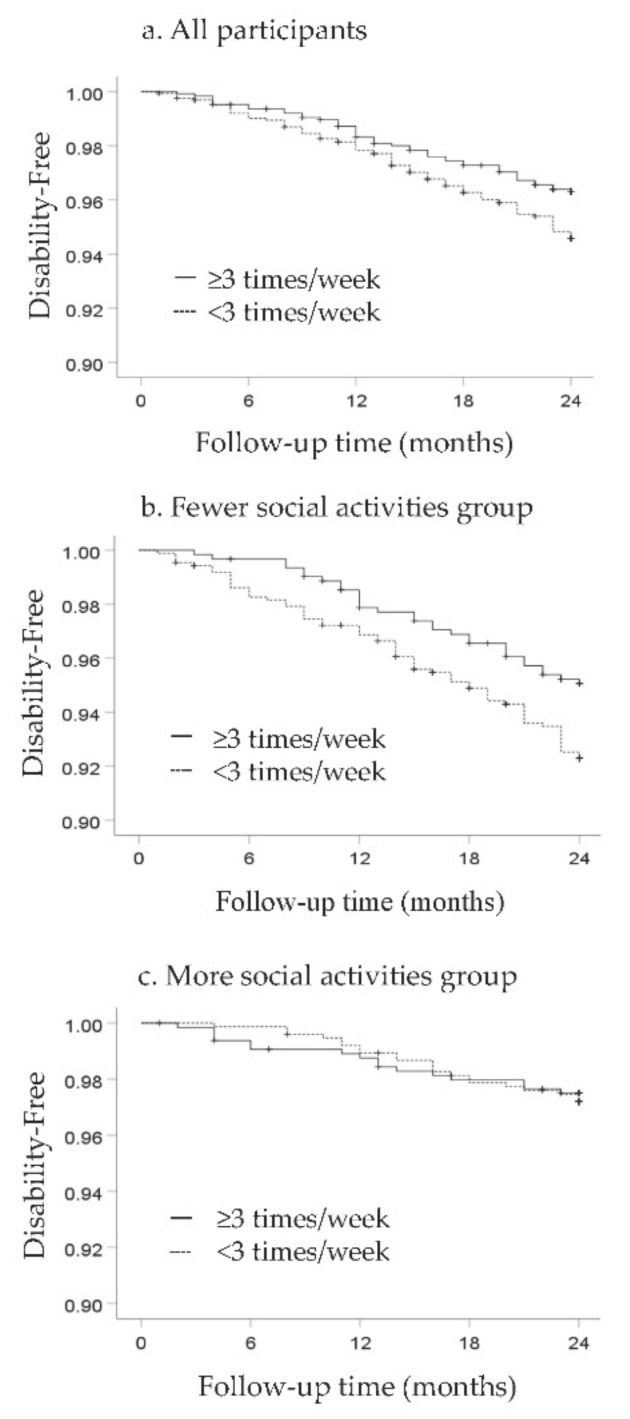
Kaplan–Meier survival estimates by walking habit and social activity groups. Those who walked ≥3 times per week had a higher probability of being disability-free than those who walked fewer than 3 times per week if their social activity was less than five activities. The relative risk reduction of disability by walking was greater among those with fewer social activities than among those with a higher number of social activities. Kaplan–Meier survival estimates by walking habit (all participants (**a**), 0–4 social activities (**b**), and 5–12 social activities (**c**)).

The incidence of disability was 19.0 per 1000 person-years for those who walked ≥3 times per week, compared with 27.9 per 1000 person-years for persons who walked <3 times per week. In Figure 1, the Kaplan–Meier survival estimates show that participants who walked ≥3 times per week had a higher probability of being disability-free than those who walked <3 times per week. The potential confounder-adjusted disability HR for participants in the regular walking group was 0.67 (CI, 0.46–0.96; *p* = 0.030).

The interaction of habitual walking and social activities was statistically significant (*p* = 0.004). In Figure 1, the Kaplan–Meier estimates show probabilities of being disability-free by walking habits among participants engaged in different social activities. The risk reduction of disability by walking was greater among participants with fewer social activities than among those with a higher number of social activities. The adjusted disability HRs by walking were 0.63 (CI, 0.40–0.98; *p* = 0.041) and 0.71 (CI, 0.36–1.38; *p* = 0.310) for persons with fewer social activities and a greater number of social activities, respectively (Table 3).

At baseline, persons who died or relocated from the study were older, took more medications, and had shorter education, lower MMSE score, lower BMI, and fewer social activities than those who were followed up and remained disability-free (*p* < 0.05). The mean number of social activity items for persons who died or relocated was 3.4 (SD, 2.3), compared with 4.8 (SD, 2.5) for those who remained in the study. Of 47 participants who died or relocated, 36 (75.0%) were involved in lower than the mean number of social activities. Of the 47 participants, 23 (48.9%) walked <3 times per week at baseline (Table 1).

## 4. Discussion

In this observational prospective cohort study of adults enrolled in a population-based cohort study, we found a reduced incidence rate of disability for persons who walked ≥3 times a week (19.0 per 1000 person-years) compared with those who walked <3 times per week (27.9 per 1000 person-years). Persons who walked ≥3 times a week had a confounder-adjusted disability HR of 0.67 (CI, 0.46–0.96) compared with those who walked <3 times per week; this corresponds to a 33% reduction in the disability risk of participants with a higher weekly walking frequency. Walking seemed to be associated with the greatest risk reduction in participants who had fewer social activities at baseline.

Habitual walking has been shown to have the potential of providing important health benefits in terms of improvement in physical performance and fitness, and prevention of physical disability in older adults [33]. New high-certainty evidence demonstrates an inverse dose–response relationship between volume of aerobic physical activity and the risk of physical functional limitations in older adults [6]. Therefore, it is recommended that as a part of their weekly physical activity, older adults should do varied multicomponent physical activity (combination of balance, strength, endurance, gait, and physical function training) at moderate or greater intensity on 3 or more days a week in order to enhance functional capacity [6]. Comparing the baseline characteristics of the participants with and without walking habits in this study, those with walking habits tended to be older, males, had more spinal diseases, a lower BMI, did not have a work, and were engaged in more social activities. Studies conducted among the older Japanese adults have shown that exercise habits and social participation were more common among older men who did not have work [11]. The difference in BMI between the groups stratified by walking habits may be related to the physiological age-related decline in body composition [34]. Age may also be an underlying factor for the higher prevalence of spinal diseases [35].

Many longitudinal studies reported that the incidence of disability was associated with the motoric cognitive risk syndrome development, physical frailty, social frailty, and low social activity levels [13,14,16,36]. As an innovative feature of our study, we found a potentially important association between walking and social activity in relation to the incidence of disability. There was a greater risk reduction of disability based on walking habits among persons with fewer social activities than those with a higher number of social activities. Fewer social activities were associated with an increased disability risk among persons who walked <3 times per week; however, this increased risk diminished among persons who walked ≥3 times per week. Our finding suggests that one of the ways that social activity may reduce the risk for disability is by modulating the relationship between walking and disability—an area worthy of additional investigation. Several longitudinal studies showed that a greater level of participation in social activities was related to a lower risk of incident functional disability [37,38]. Persons who participate in social activities have an opportunity for communicating and collaborating with, and helping others [39]; therefore, the risk of developing disability may have been reduced in the group that performed many social activities, even if they walked <3 times per week. These results suggest the importance of participating in many group activities with two or more people. This supports the findings of previous studies that social activities reduce the risk of incident functional disability [37,38].

In the present study, we added the Walk Score, which is associated with the number of steps per day, minutes of leisure walking, and daily moderate- to vigorous-intensity physical activities [27,28,29], to the covariates; however, future studies should add a method for the accurate measurement of the actual walking intensity and duration. We used the LTCI system for disability incidents. It has been used in many previous studies [14,16,31,32]. However, it has also been pointed out that there is a risk that the incidence of disability may be missed due to social factors, such as the lack of encouragement from the surrounding community [30]. This point needs to be considered in future studies.

The strengths of this study include the large sample size and social activity assessment. To our knowledge, this is the first study that evaluated walking habits and participation in social activities to examine the relationship between participation in social activities and disability incidence. Before the COVID-19 pandemic, previous studies reported that 1 in 4 older adults was socially isolated and more than 40% experienced loneliness [40]. Decades of observational studies have demonstrated the long-term negative health outcomes of social isolation and loneliness [41,42]. The COVID-19 crisis has exacerbated these challenges, with worsening social isolation and loneliness among those who live alone or are frail and even declines in the well-being of older adults with previously active or healthy social lives [40]. The results of this study suggest one way to prevent an increase in the incidence of disability after the convergence of COVID-19. This means that not only acquiring the habit of walking but also participating in social activities in groups of two or more people may increase the possibility of preventing disability in the older adults. However, this questionnaire is based on self-report. Social activity and physical activity are known to be highly correlated [43]. Therefore, it is possible that there was a bias between both activities. In the future, it will be necessary to conduct research using objective indicators, such as walking time, exercise intensity, and the amount of conversation with others in social activities to improve the effect of preventing disability, and we are proceeding with our study plan. However, this study also has some limitations. First, we did not use random sampling for data collection; hence, there was a possibility of disability incidence under-reporting among older adults. The participants in our sample were all capable of accessing health checkups from their homes, implying that people with various other conditions were excluded. Second, we did not address other covariates related to health variables (e.g., smoking and alcohol use) that could also affect cumulative age-related changes; therefore, future studies should include such variables. Third, we have not been able to clarify the effect of participation in incidental physical activities associated with social activities. In addition, the walking frequency/habits in this study were self-report. Furthermore, it is possible that they may have engaged in activities that were not listed. To objectively measure interaction with others and physical activity, we are preparing a study to measure the amount of conversation and physical activity in the future. Finally, we did not have a good method of measuring the walking intensity; thus, future studies are required to investigate the dose-versus-threshold-based association between walking and incidence disability.

Habitual walking was associated with disability incidence, with a more pronounced effect among older adults who were less likely to engage in social activities. Given the increasingly high prevalence of disability, its strong association with numerous adverse health outcomes, clinicians can focus more on considering an older adult’s walking frequency and participation in social activities in their day-to-day practice in adult-centered care; this, in turn, may lead to better outcomes in primary disability prevention.

## Figures and Tables

**Table 1:** Baseline characteristics of the study participants by follow-up status.

Variable	Total	Participants Free of Disability	Participants with Disability	Participants Who Died or Relocated	*p*-Value	Post Hoc
	(*n* = 2873)	(*n* = 2693)	(*n* = 133)	(*n* = 47)		
Mean age at baseline, years	73.1 ± 5.9	72.7 ± 5.7	80.2 ± 5.8	76.8 ± 7.3	<0.001 *	Free < Died or relocated <Disability
Female sex, number (%)	1618 (56.3)	1518 (56.4)	78 (58.6)	22 (46.8)	0.364	
Medication use, number	2.9 ± 2.6	2.8 ± 2.5	4.1 ± 2.6	4.0 ± 3.2	<0.001 *	Free < Disability, Died or relocated
Chronic disease						
Heart disease, no (%)	2408 (83.8)	2262 (84.0)	106 (79.7)	40 (85.1)	0.410	
Diabetes, no (%)	2467 (85.9)	2315 (86.0)	113 (85.0)	39 (83.0)	0.805	
Hyperlipidemia, no (%)	1990 (69.3)	1857 (69.0)	97 (72.9)	36 (76.6)	0.345	
Spinal diseases, no (%)	2320 (80.8)	2182 (81.1)	105 (78.9)	33 (70.2)	0.145	
Cohabitation status, no (%)	341 (11.9)	308 (11.4) ^§^	24 (18.0) ^‡^	9 (19.1)	0.021	
Years of education, years	11.0 ± 2.3	11.1 ± 2.3	10.0 ± 2.3	10.2 ± 2.7	<0.001 *	Disability, Died or relocated < Free
Physical function						
Grip strength, kg	27.3 ± 7.6	27.5 ± 7.6	23.5 ± 6.8	26.5 ± 7.1	<0.001 *	Disability < Free
Cognitive function						
Mini-Mental State Examination score	27.0 ± 2.7	27.1 ± 2.5	24.8 ± 3.4	25.2 ± 3.8	<0.001 *	Disability, Died or relocated < Free
Body mass index, kg/m^2^	23.5 ± 3.3	23.5 ± 3.3	23.1 ± 3.9	22.9 ± 4.0	0.330	
Metabolic parameters						
Total serum protein, g/dL	7.4 ± 0.4	7.5 ± 0.4	7.4 ± 0.5	7.4 ± 0.6	0.253	
Glycated hemoglobin, %	5.8 ± 0.7	5.8 ± 0.6	5.8 ± 0.7	5.8 ± 0.6	0.955	
Walk score	67.9 ± 11.6	68.0 ± 11.6	67.0 ± 13.0	67.3 ± 11.5	0.665	
Participants who walked ≥3 times per week	1255 (43.7)	1186 (44.0)	46 (34.6) ^§^	23 (48.9)	0.077	
Social activity						
Officer of a senior club or neighborhood association, yes (%)	799 (27.8)	758 (28.1)	32 (24.1)	9 (19.1)	0.242	
Attending a regional event, yes (%)	653 (22.7)	628 (23.3) ^‡^	17 (12.8) ^§^	8 (17.0)	0.012 ^†^	
Engage in environmental beautification activities, yes (%)	779 (27.1)	735 (27.3)	35 (26.3)	9 (19.1)	0.450	
Teaching, yes (%)	263 (9.2)	254 (9.4) ^‡^	8 (6.0)	1 (2.1)	0.099	
Supporting activity, yes (%)	430 (15.0)	413 (15.3) ^‡^	10 (7.5) ^§^	7 (14.9)	0.048	
Working, yes (%)	900 (31.3)	874 (32.5) ^‡^	16 (12.0) ^§^	10 (21.3)	<0.001 ^†^	
Go to karaoke, yes (%)	632 (22.0)	598 (22.2)	30 (22.6)	4 (8.5) ^§^	0.079	
Eating out or tea party with friends, yes (%)	2232 (77.7)	2111 (78.4) ^‡^	93 (69.9) ^§^	28 (59.6) ^§^	0.001 ^†^	
Go shopping with a friend, yes (%)	1375 (47.9)	1308 (48.6) ^‡^	55 (41.4)	12 (25.5) ^§^	0.002 ^†^	
Talk to a friend (including phone), yes (%)	2616 (91.1)	2457 (91.2)	117 (88.0)	42 (89.4)	0.401	
Attending an event or concerts, yes (%)	1454 (50.6)	1399 (51.9) ^‡^	39 (29.3) ^§^	16 (34.0) ^§^	<0.001 ^†^	
Go traveling, yes (%)	1337 (46.5)	1287 (47.8) ^‡^	37 (27.8) ^§^	13 (27.7) ^§^	<0.001 ^†^	
Social activity, items	4.7 ± 2.5	4.8 ± 2.5	3.7 ± 2.4	3.4 ± 2.3	<0.001 ^†^	Disability, Died or relocated < Free

* *p*-values reported from one-way analysis of variance. Significant differences were determined by Tukey post-hoc test. ^†^ *p*-values were determined by Pearson’s chi-square test. ^‡^ Statistically significant association was determined by adjusted standardized residual >1.96 (*p* < 0.05). ^§^ Statistically significant association was determined by adjusted standardized residual <−1.96 (*p* < 0.05).

**Table 2:** Baseline characteristics of the study participants by walking levels.

Variable	Participants Who Walked ≥3 Times per Week	Participants Who Walked <3 Times per Week	*p*-Value
	(*n* = 1255)	(*n* = 1618)	
Mean age at baseline, years	73.4 ± 5.6	72.9 ± 6.2	0.012 *
Female sex, number (%)	643 (51.2) ^§^	975 (60.3) ^‡^	<0.001 ^†^
Medication use, number	3.0 ± 2.5	2.9 ± 2.5	0.390
Chronic disease			
Heart disease, no (%)	1044 (83.2)	1364 (84.3)	0.421
Diabetes, no (%)	1074 (85.6)	1393 (86.1)	0.694
Hyperlipidemia, no (%)	867 (69.1)	1123 (69.4)	0.833
Spinal diseases, no (%)	1036 (82.7) ^‡^	1284 (79.4) ^§^	0.027 ^†^
Cohabitation status, no (%)	142 (11.3)	199 (12.3)	0.418
Years of education	11.1 ± 2.4	11.0 ± 2.3	0.308
Physical function			
Grip strength, kg	27.8 ± 7.6	26.8 ± 7.5	0.270
Cognitive function			
Mini-Mental State Examination score	26.9 ± 2.7	27.0 ± 2.6	0.148
Body mass index, kg/m^2^	23.3 ± 3.1	23.6 ± 3.5	0.002 *
Metabolic parameters			
Total serum protein, g/dL	7.4 ± 0.4	7.4 ± 0.4	0.923
Glycated hemoglobin, %	5.8 ± 0.7	5.8 ± 0.6	0.592
Walk score	68.0 ± 11.6	68.0 ± 11.7	0.960
Social activity			
Officer of a senior club or neighborhood association, yes (%)	401 (32.0) ^‡^	398 (24.6) ^§^	<0.001 ^†^
Attending a regional event, yes (%)	320 (25.5) ^‡^	333 (20.6) ^§^	0.002 ^†^
Engage in environmental beautification activities, yes (%)	389 (31.0) ^‡^	390 (24.1) ^§^	<0.001 ^†^
Teaching, yes (%)	141 (11.2) ^‡^	122 (7.5) ^§^	0.001 ^†^
Supporting activity, yes (%)	205 (16.3)	225 (13.9)	0.070
Working, yes (%)	326 (26.0) ^§^	574 (35.5) ^‡^	<0.001 ^†^
Go to karaoke, yes (%)	315 (25.1) ^‡^	317 (19.6) ^§^	<0.001 ^†^
Eating out or tea party with friends, yes (%)	973 (77.5)	1259 (77.8)	0.857
Go shopping with a friend, yes (%)	601 (47.9)	774 (47.8)	0.978
Talk to a friend (including phone), yes (%)	1151 (91.7)	1465 (90.5)	0.276
Attending an event or concerts, yes (%)	673 (53.6) ^‡^	781 (48.3) ^§^	0.004 ^†^
Go traveling, yes (%)	613 (48.8) ^‡^	724 (44.7) ^§^	0.029 ^†^
Social activity, number	4.9 ± 2.6	4.6 ± 2.4	0.001 *

* *p*-values reported from unpaired *t*-test. ^†^ *p*-values were determined by Pearson’s chi-square test. ^‡^ Statistically significant association was determined by adjusted standardized residual >1.96 (*p* < 0.05). ^§^ Statistically significant association was determined by adjusted standardized residual < −1.96 (*p* < 0.05).

**Table 3:** Cox regression analysis of the relationships between walking habit and incidence of disability in each social activity group.

All Participants	Number of Participants	Incident Disability Rate	Model 1 (Crude Model)			Model 2			Model 3		
			HR	95% CI	*p*	HR	95% CI	*p*	HR	95% CI	*p*
Participants who walked <3 times per week	1255	46 (3.7%)	1.00			1.00			1.00		
Participants who walked ≥3 times per week	1618	87 (5.4%)	0.68	0.47–0.97	0.032	0.70	0.49–1.00	0.050	0.67	0.46–0.96	0.030
Fewer social activities group (0–4 activities)	Number of participants	Incident disability rate	Model 1 (Crude Model)			Model 2			Model 3		
			HR	95% CI	*p*	HR	95% CI	*p*	HR	95% CI	*p*
Participants who walked <3 times per week	613	30 (4.9%)	1.00			1.00			1.00		
Participants who walked ≥3 times per week	866	66 (7.6%)	0.63	0.41–0.97	0.037	0.68	0.44–1.06	0.086	0.63	0.40–0.98	0.041
More social activities group (5–12 activities)	Number of participants	Incident disability rate	Model 1 (Crude Model)			Model 2			Model 3		
			HR	95% CI	*p*	HR	95% CI	*p*	HR	95% CI	*p*
Participants who walked <3 times per week	642	16 (2.5%)	1.00			1.00			1.00		
Participants who walked ≥3 times per week	752	21 (2.8%)	0.90	0.47–1.72	0.737	0.78	0.41–1.51	0.466	0.71	0.36–1.38	0.310

Model 1 is a crude model. Model 2 is adjusted for age and sex. Model 3 is adjusted for the covariates in Model 2 and years of education, heart disease, diabetes, hyperlipidemia, spinal diseases, cohabitation status, BMI, total serum protein, HbA1c, medication, MMSE score, grip strength, and walk score. CI: confidence interval; HR: hazard ratio; BMI, body mass index.

## Supplementary Materials

The following are available online at https://www.mdpi.com/article/10.3390/jcm10091895/s1, Table S1: Frequency of physical activities.
