# Supplementary material for: Participation in Social Activities and Relationship between Walking Habits and Disability Incidence
Source: J Clin Med. 2021 Apr 27;10(9):1895. doi: 10.3390/jcm10091895 (PMC8123784; doi:10.3390/jcm10091895)
Supplement: Supplementary file 1 [file jcm-10-01895-s001.zip › jcm-1165614-supplementary.pdf]

Table S1. Frequency of physical activities.

	Participants who exercised ≥3 times per week	Participants who exercised <3 times per week
Walking, n (%)	1255 (43.7)	1618 (56.3)
Cycling, n (%)	380 (13.2)	2493 (86.8)
Jogging, n (%)	208 (7.2)	2665 (92.8)
Swimming, n (%)	30 (1.0)	2843 (99.0)
Muscle training, n (%)	272 (9.5)	2601 (90.5)
Yoga, n (%)	19 (0.7)	2854 (99.3)
Gymnastics, n (%)	803 (27.9)	2070 (72.1)
Dancing, n (%)	39 (1.4)	2834 (98.6)
Hiking, n (%)	2 (0.1)	2871 (99.9)
Playing golf, n (%)	41 (1.4)	2832 (98.6)
Playing grand golf, n (%)	96 (3.3)	2777 (96.7)
Ball exercise, n (%)	72 (2.5)	2801 (97.5)
